# A national framework for transition to precision medicine

**DOI:** 10.3389/fmed.2024.1396496

**Published:** 2025-01-27

**Authors:** Samaneh Karimi Esboei, Sepehr Ghazinoory, Fatemeh Saghafi

**Affiliations:** ^1^Faculty of Technology & Industrial Management, College of Management, University of Tehran, Tehran, Iran; ^2^Department of Information Technology Management, Tarbiat Modares University, Tehran, Iran

**Keywords:** precision medicine, personalized medicine, stratified medicine, transition framework, socio-technical transition, transition pathway

## Abstract

Precision medicine (PM) is transforming healthcare by offering tailored interventions that address individual variability, transforming patient care and outcomes. PM is based on providing health-oriented services according to genetic characteristics, individual and family medical history, lifestyle, place of residence, and other personalized characteristics. This study aims to establish an appropriate framework for implementing PM in Iran. First, the global transition framework to PM was drawn by a systematic review, and then a framework for transition to PM in Iran was drawn by a case study through semi-structured interviews, an expert panel, and an analytic hierarchy process (AHP) questionnaire. The statistical sample of the study comprised PM specialists, researchers, and patients whose PM plays a significant role in their diagnosis and treatment. The sampling method was non-random with a combination of purposive and snowball techniques. The results from the systematic review show that for the transition to PM, we must first move from common medicine to stratified medicine and then PM. Moving toward PM requires strong economic, social, political, institutional, industrial, and, most importantly, technological infrastructures. These infrastructures will vary from country to country. In general, coexistence between the health system and PM technologies did not exist in the beginning, but it will emerge with its development. The resistance of the health system to accepting PM will gradually decrease. Furthermore, the government plays a key role in the early phases, while market and PM demand become more prominent during the development. New health actors will also develop PM, and out-of-date actors will be deleted or replaced. But moving toward PM is slightly different in Iran, particularly in the middle phases of transition.

## 1 Introduction

It is expected that emerging or new technologies will transform the world in the coming years. Countries that are more receptive to new knowledge and technologies are likely to have a more dynamic and developed economy. In the near future, we will witness fundamental changes in medicine (1). One of the most important changes in medicine is the development of precision medicine (PM), which means providing individual-oriented health services (2).

PM has been widely applied to oncology, genetic diseases, pharmacogenomics, non-invasive prenatal testing, genetic risk, and public health. In the world, the applications of PM are rapidly growing nowadays, which makes great efforts in lifesaving, genetic risk warning, healthcare cost reduction, and quality of life improvement. The use of PM technologies and applications has developed in many countries, such as the US^1^, Canada^2^, Australia^3^ (3), the UK,^4^ and EU^5^. Precision medicines are prescribed in more than 50% of all prescriptions in the EU (4). Many developing countries, such as Africa, Singapore^6^, Saudi Arabia^7^, India^8^, Qatar, and Iran, are also launching biobanks as the first step for implementation of PM (5–7).

The emergence of new medical technologies, especially in relation to the human genome, has facilitated this process. Therefore, the transition from the common way of providing medical services to the new way, i.e., PM, is a living and ongoing phenomenon. In the near future, the transition from population-oriented medicine to PM will be a requirement. The concept of transition has its roots in biology and population dynamics. In recent times, the process has typically been sparked by improvements in hygiene and healthcare. A transition can be defined as a gradual, continuous process of change in which the structural character of a society (or a complex sub-system of society) transforms (8). Therefore, moving toward PM is a kind of socio-technical transition in medicine.

Identifying the appropriate transition pathway to PM and prescribing it actually leads to removing this socio-technical system from the locked situation so that with its proper management, desirable social and health goals can be better fulfilled. So, what is the appropriate framework for moving to PM? This study has developed a framework for the transition to PM through a systematic review and a case study. The proposed framework addresses existing gaps in PM’s development by identifying the characteristics of transition paths toward PM and predicting the requirements, infrastructures, and challenges of each phase of PM development. Eventually, these lead to the right decision-making by policymakers for PM’s development. This is followed by a literature background, research gap, methods, results, discussion, and conclusion.

## 2 Literature background

### 2.1 Precision medicine

Treatment of patients is currently based on traditional frameworks, i.e., disease pathology, its diagnosis based on related symptoms, and the same protocols for almost everyone. In other words, the care guidelines or medication for a certain disease are somewhat the same for everyone. Recent advances in providing medical services called precision medicine show that using a care guideline or medication for patients with a specific disease is not only costly and ineffective, but also it may cause side effects for a group of the patients. PM is an evolutionary process in a continuous framework over time. Precision medicine and personalized medicine are conceptually very close to each other, and the context of countries determines the use of the term personalized medicine or precision medicine (9).

Stratified medicine is often considered a synonym for both personalized and precision medicine, but these three terms are also related to distinct facets of treatment and care. Stratified medicine is a term that has been widely used since the 1990s in relation to genomics and subsequently other fields of biology. It is a form of medicine that sorts a population into the most biologically appropriate groupings to determine the optimal therapeutic response, but precision medicine builds on the finer subclassification of disease to add repeated monitoring of disease markers to enable recursive tailoring of treatment to individual response (10).

Stratified medicine is “an approach to therapy that forms a key step on the path toward personalized healthcare, not personalized medicine, because each one describes expectations about the future of medicine and healthcare that data-intensive innovation promises to bring forth. Each one reflects different views of what the aims of emerging biomedical research modes are and should be in the context of healthcare and positions patients and citizens differently with respect to health and biomedicine as well as in relation to governance and the state (11).

According to PM, some patients may not even need medication due to their biomarkers, medical history, genetic characteristics, and lifestyle, and they can recover with preventive measures. In this way, there will be no additional financial burden for the health system.

It is important to pay attention to PM and identify its transition pathways from several dimensions:

1. It will prevent the wastage of public resources, especially health resources, because it will prevent the medication or ineffective treatment procedures. For example, ten of the most effective drugs in the United States are useful for only 1 in 25 people and 1 in 4 people. Worse, the side effects and adverse reactions that make these drugs wrong. 30% of hospital admissions per year are caused by the wrong drugs. Some drugs, such as statins—routinely used to lower cholesterol—are effective for only one out of 50 people (12). Furthermore, people of non-European ancestry are critically underrepresented in medical and genomic research; therefore, some medicines may not be useful for these people (13). Kasturba et al., in a scoping review, show that the majority of PM studies have concluded that PM intervention was at least cost-effective compared to usual care (14). Moreover, four economic case studies for PM were reviewed, which show Potential sources of value in PM studies include improved clinical effectiveness, reduced adverse drug reactions, improved health outcomes, and/or reduced healthcare resource use compared to usual care, and improved diagnostic accuracy and potentially reduced healthcare resource use (15).
2. PM is based on biotechnologies, especially in the pharmaceutical industry, from initial development to market models, and will change the entire value chain of the pharmaceutical industry.^9^ According to the recent report published by the PM Coalition, more than 34% of all the new drugs approved by the US FDA in 2022 are personalized medicines (52).^10^
3. PM is used in diagnosis and care services, nutrition and individual well-being, stem cell therapy, omics sciences, and treatment of many diseases, including central nervous system (CNS) diseases, respiratory diseases, and diabetes. Also, PM has played a significant role in finding prevention and treatment guidelines during the control of the epidemic of Covid-19 (16, 53).^11^

On the other hand, the fifth industrial revolution is also evolving, and it is focused on personalization, sustainability, and human-machine cooperation. In other words, all industries are moving toward personalization by relying on artificial intelligence (AI), including the health sector (17). That is, in the health sector, AI helps doctors to choose the best and most unique treatment or diagnostic measures for a person according to the biological characteristics, lifestyle, nutrition, medical history, place of residence, genetic sequence, etc.

So the framework of 21st century medicine is to move from population-centered to person-centered, or personalized, medicine (2).

Therefore, according to the importance and necessity of PM mentioned above, it appears that the medicine of the 21st century is precision medicine. But PM, like any new technological and innovative paradigm, is not automatically absorbed and developed but is influenced by the economic, social, and institutional infrastructure of countries. For instance, the capacity for absorption of new PM technologies in the UK is different from that of developing countries like Iran. There are different pathways to absorb and develop new technologies according to the economic, social, institutional, technological, environmental, and political infrastructures. These are called socio-technical transition pathways. So in order to absorb and develop PM (an emerging technological paradigm in health), we also need to go through the path/paths of socio-technical (ST). Now what is the ST?

### 2.2 Socio-technical transitions

Transitions are long-term transformation processes (usually 25–50 years) in which society changes fundamentally over several decades or generations (1). They are the result of the co-evolution of technological, institutional, cultural, ecological, and economic transformations at different levels (1, 8, 18). A transition can be defined as a gradual, continuous process of change where the structural character of a society (or a complex sub-system of society) transforms^12^. ST occurs when the dominant structural characteristics in society (regimes) are under pressure due to external changes as well as endogenous innovation (19).

**Figure 1:**
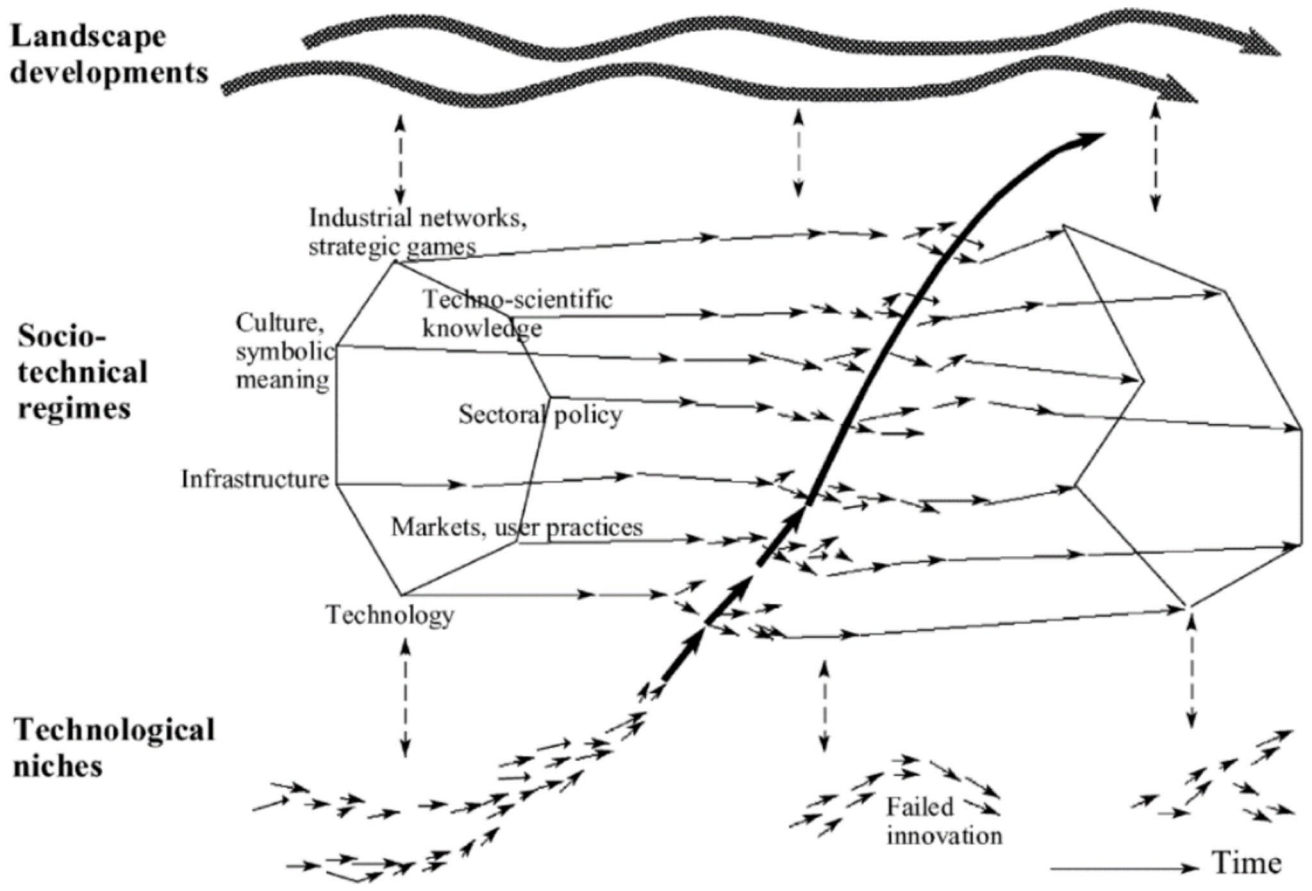
ST pathways based on the S curve (transition phases) [Geels, 2002; (22)].

Transition consists of four phases, which are pre-development, takeoff, acceleration, and stabilization. These phases are common in all TPs. Transition pathways are a form of socio-technical scenario that seek the potential future development of socio-technical systems through interactions between moving processes at the three levels, including niche, regime, and landscape (20, 21). According to the multi-level perspective (MLP), transitions are formed from the interaction between processes at these three levels: (1) niche innovations that cause movement; (2) changes in landscape that exert pressure on the regime; and (3) the instability of the regime that creates windows of opportunity for niche innovations. The interactions between multilevel perspective (MLP) and transition phases are seen in Figure 1 (22).

- Niches (incubation spaces) are the microlevel of this model, and radical innovations occur in them. Innovation is weak in this phase, so it must be formed in supported areas by the mainstream of market choice. These areas are important because they create learning space (54).
- ST regimes are the middle level of this model. The ST regime is defined as a collection of common, relatively sustainable, and consistent rules that lead the behavior of actors in a specific system. These rules are embedded in different elements of the socio-technical system and form innovative activities toward a specific direction of gradual innovation (for example, increasing the fuel efficiency of cars) (23).
- At the landscape level (macro level), there is a set of context variables, such as material infrastructure, political conditions and culture, social values, paradigms, macroeconomics, demography, and the environment, which they change independently and very slowly and affect the transition processes (8). Changes in the landscape exert significant pressure on the current regime (55).

Common TP include transformation, reconfiguration, technological replacement, and de-alignment and re-alignment (23–25, 27).

#### 2.2.1 Transformation pathway

**Figure 2:**
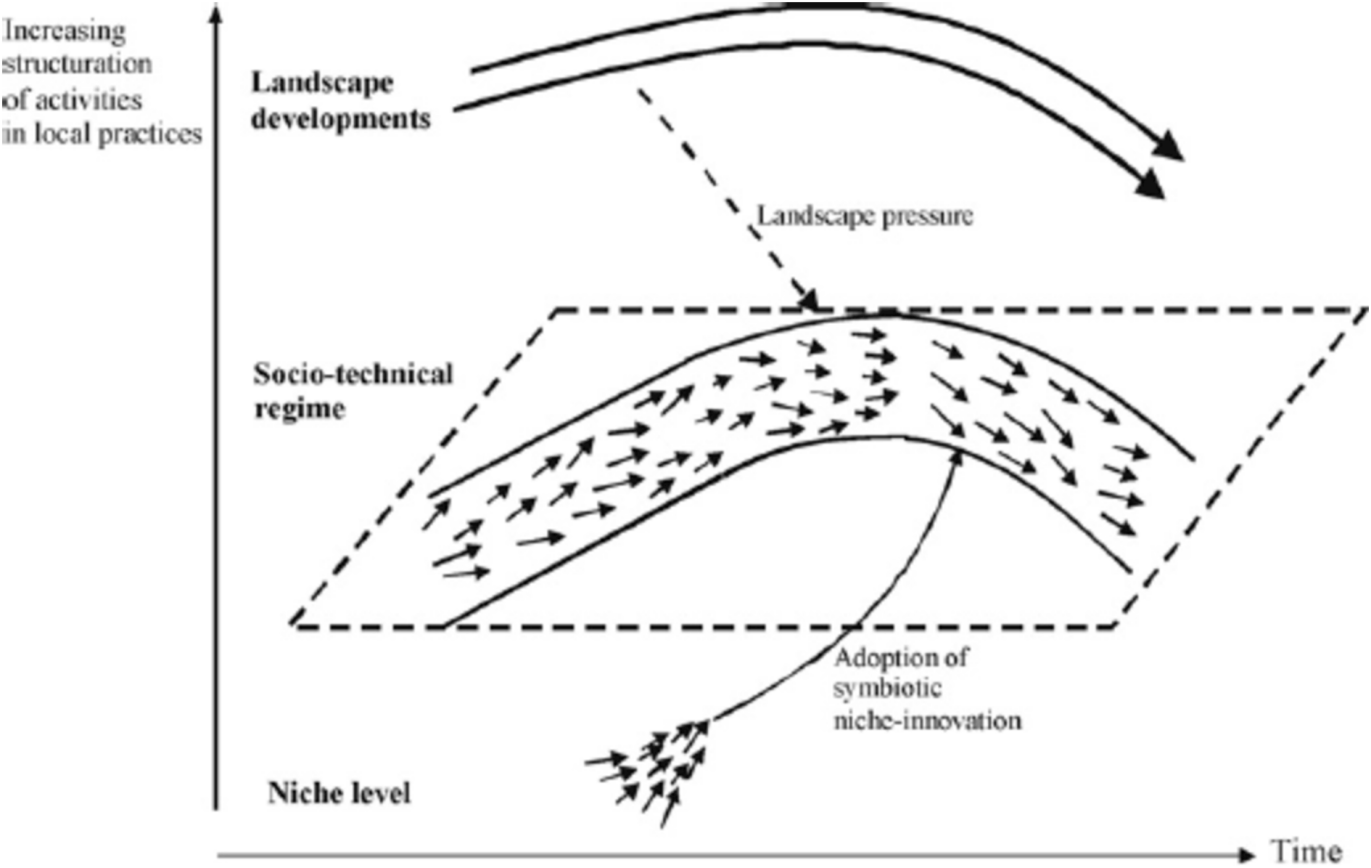
Transformation pathway (Source: (23).

In this path, processes of change arise from the interaction of an evolving landscape with the socio-technical regime (but not with the technological niche level). A transformation path takes place when landscape pressures are moderate and niche innovations have not yet sufficiently developed. e.g., the transition from cesspool to sewer system in the Netherlands (25). In this path, resistance of the regime is very high, and government intervention can be used to focus and encourage the speed of change (26, 27) (Figure 2).

#### 2.2.2 Reconfiguration pathway

**Figure 3:**
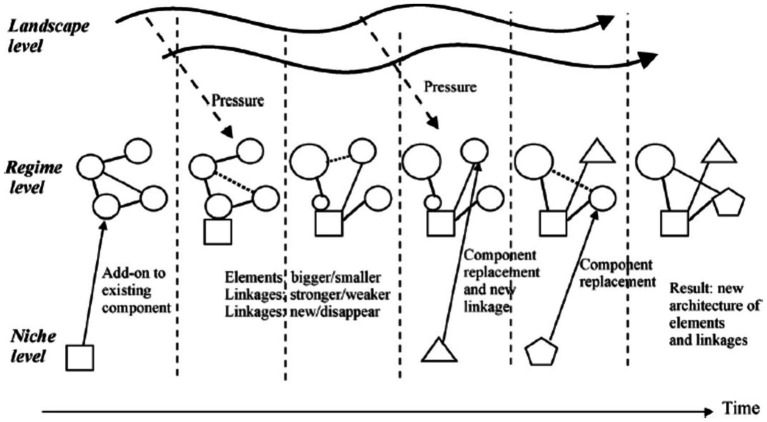
Reconfiguration pathway (Source: (23).

It is the result of interactions between all three levels in MLP. In this path, niche innovations are more developed when regimes face problems and external landscape pressures. In response, the regime adopts certain niche innovations into the system as add-ons or component substitutions, leading to a gradual reconfiguration of the basic architecture and changes in some guiding principles, beliefs, and practices. The new regime also grows out of the old regime. It differs from the transformation path in that the cumulative adoption of new components changes the basic architecture of the regime substantially. The main interaction is between regime actors and niche actors, who develop and supply the new components and technologies (26, 27). In this path, the landscape does not seem to play a prominent role (e.g., the transition from traditional industrial production to mass production in the US) (25) (Figure 3).

#### 2.2.3 Technological substitution pathway

**Figure 4:**
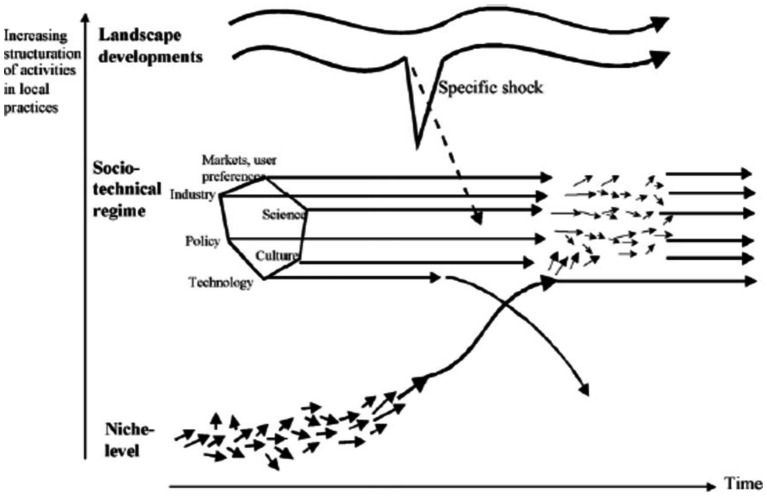
Technological substitution pathway (Source: (23).

Replacement of one dominant technology within the socio-technical regime by another as a consequence of interaction between all three levels (26). It is the result of interactions between all three levels in MLP. Geels and Schot use the term “technological substitution” when there are large landscape changes, and they produce problems and tensions in regimes while niche innovations have sufficiently developed and are ready to break through (e.g., the introduction of the steamboat as a replacement for the sailing ship in the UK) (25). In this pathway, newcomers (niche actors) compete with incumbent regime actors (27) (Figure 4).

#### 2.2.4 De-alignment and re-alignment pathway

Interaction between the three levels resulting in competition between a dominant technology within the regime and a number of other competing options that have different performance characteristics, eventually resolved through emergence of a new dominant option (new actors, guiding principles, beliefs, and practices) (26, 27).

**Figure 5:**
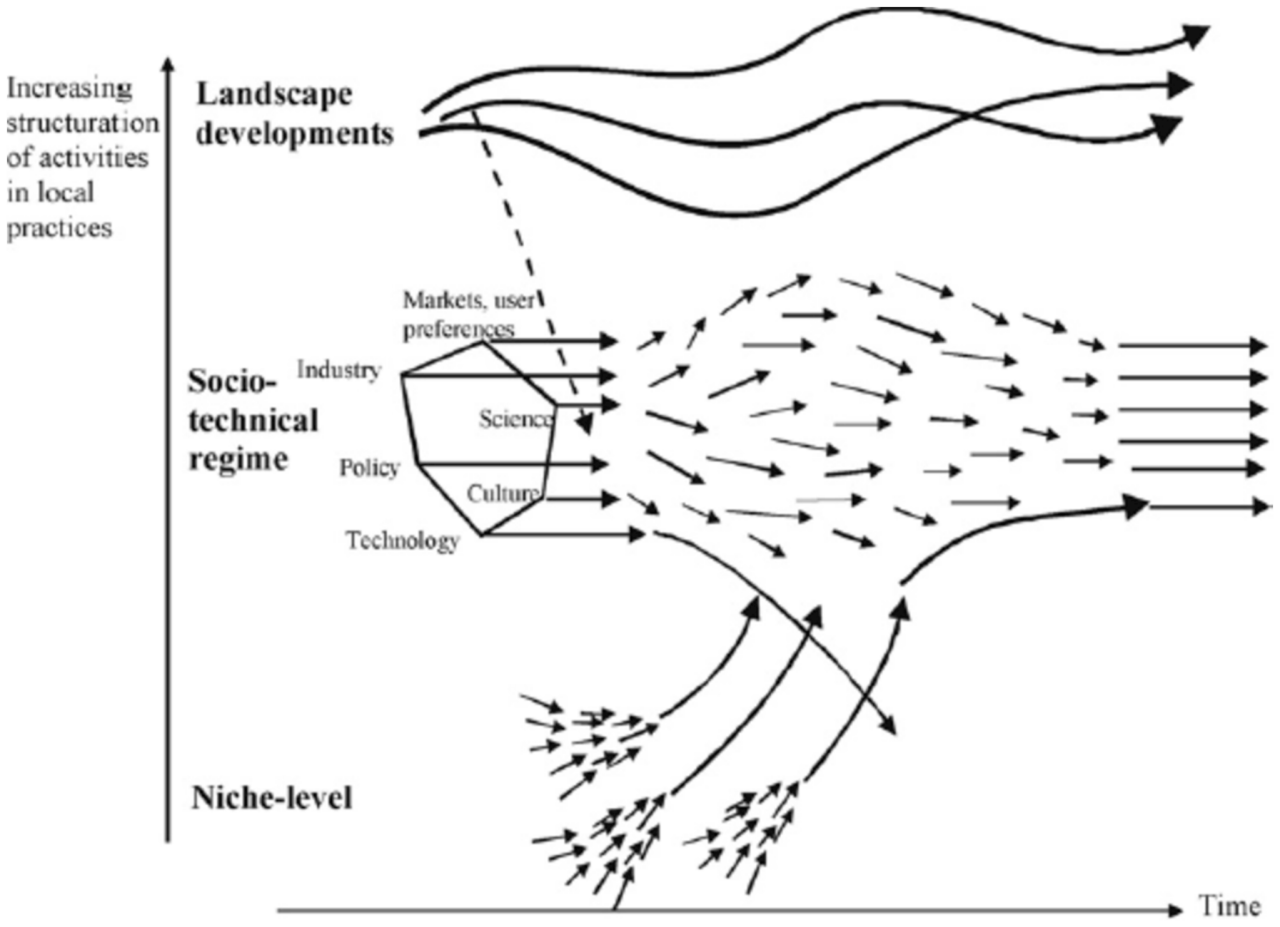
De-alignment and re-alignment pathway (Source: (23).

Major, divergent, sudden, and large landscape changes lead to huge problems in the regime, while none of the niche innovations are sufficiently developed yet. This leaves space for multiple innovations to emerge until one eventually dominates and forms a new core for the regime (e.g., the transition from horse-drawn carriage to automobile in the US). The regime experiences major internal problems, collapses, erodes, and de-aligns, and regime actors lose faith in the future of the system. This pathway is therefore more dependent on external developments and/or strong policy interventions (25, 27) (Figure 5).

In addition to these four pathways, in some studies, a sequence of transitions is also mentioned, which is more of a theoretical structure.

If landscape pressure takes the form of ‘disruptive change’, a *sequence of transition pathways* is likely, beginning with transformation, then leading to reconfiguration, and possibly followed by substitution or de-alignment and re-alignment. ‘Disruptive change’ is a specific kind of landscape development. Because of its slow speed, actors initially perceive only moderate change. As pressure continues to build in a certain direction, landscape change gradually becomes more disruptive. This characteristic may lead to a particular sequence of transition pathways. This sequential pattern indicates that crossovers may occur between transition pathways (23).

Two types of sequence of transitions have been identified and repeated more than the others, which Kanger (28) defined as “transition in limbo”: (1) shared transition pathway, that is, De-alignment and re-alignment + Technological substitution sequentially or simultaneously and (2) broad transformation + reconfiguration simultaneously (28–31).

The characteristics of ST pathways [(20, 24, 25, 27, 28, 30, 32–39) are listed in Table 1.

**Table 1:** The characteristics of ST pathways.

Transition path	landscape pressure	Resilient/Fragile regime	Maturity of niche technology	Degree of change	Degree of changes in regulations & institutions	Pattern governance or action space	Transition mechanism	Niche-regime co-existence	Incumbent actors’ fate after transition	Importance of value change	Co- ordination	MLP levels involved
Transformation	Moderate, spread disruptive	Resilient	Low (emergent)	Incremental - modular (no change in regime architecture)	Moderate (limited radical change)	Market-led+ civil society	Absorbing and personalizing niche technologies	Present	Majority	Demand is more important	Low	Landscape & regime
Technological substitution	Very high, avalanche	Fragile	Full (mature)	radical - architectural	Very high	Market-led	Clustering and empowering of niches & Absorption of external innovators	Present	Minority	Supply is more important	High	Landscape, regime & niche
Reconfiguration	High, Moderate, disruptive	Resilient	Moderate (mature)	Incremental - modular (in the main system architecture)	High	Strong government involvement+ market led	Absorbing and personalizing niche technologies	Present	Majority/ minority/ extinction	Balance between supply & demand	Moderate	Landscape, regime & niche
De-alignment and re-alignment	Unbearable, avalanche	Fragile	Moderate (emergent)	Radical- architectural	Very high	Government+ civil society	Clustering and empowering of niches & Absorption of external innovators	Absent	Extinction	Supply is more important	Very high	Landscape, regime & niche
De-alignment and re-alignment + substitution	Moderate, specific shock	Fragile	Low (emergent)	Radical technological change	High (creation of new laws & institutions)	Government	Reinforcing Incumbent regime, creating new markets	Absent	Minority	Supply is more important	High	Landscape, regime & niche
Broad transformation + reconfiguration	Moderate, specific shock	resilient	Low (emergent)	Incremental - architectural	Incremental - Institutional changes	Government+ market (bold role of the market)	Absorbing and personalizing niche technologies	Present	Majority	Demand is more important	low	Landscape, regime & niche

### 2.3 Research gap

The transition from common medicine to PM is an ongoing phenomenon, and in the near future, transition from common medicine (population-oriented) to PM is inevitable. Therefore, recognizing the appropriate framework and TPs for PM and adopting appropriate policies for the development of related technologies before their growth in society and the creation of related challenges can be very helpful. The concept of transition has its roots in biology and population dynamics (8), and recently, this process has started with a spark in health services. While the systematic review shows that the studies about ST pathways (most of them conducted by Geels and Hekkert) (24, 40, 41) were mostly in the field of renewable energy, there is no study that particularly deals with the transition to PM or the transition framework for health sector.

Furthermore, the researches that have been carried out so far on PM have been cross-sectional and non- comprehensive, this means that PM has been studied more from specialized, clinical and technological perspective (2, 12, 16, 42), and about policymaking for PM has been discussed very superficially and cross-sectionally and indirectly in some studies, and so far, a comprehensive model for Transition to PM is not provided.

The study intends to connect the theory of TPs (considering characteristics of transition paths and transition phases) to the presented cross-sectional patterns for PM and provide a comprehensive framework for moving to PM. Thus, there is a research gap in management and transition to PM. Research about the gap helps policymakers make decisions about how to move toward PM and development of its technologies. The research results can also be prescribed for countries’ health systems that are moving toward PM.

Another theoretical contribution is using ST theory to prescribe and with a futuristic attitude, while the previous transition studies were retrospective and descriptive. The previous transition studies have mainly been case studies about transitions of energies and transportation systems that occurred in developed countries and retrospectively described and analyzed the paths of energy transition, transportation, etc. For example, the transition from fossil fuels to wind and renewable energies during the past 20, 30 or 50 years. But in the study, we used the theory of transition to predict the transition paths to PM in order to use the obtained framework to prescribe future PM policies in the health sector.

Since PM has not yet been developed in different countries of the world, including Iran, using a cohesive framework for its development and prescription in the future is essential. It can help health policy makers to predict the necessary infrastructure and PM challenges in different phases of PM development and make appropriate decisions and policies.

In the study, such a framework has been obtained by integrating the ST theory (one of the important theories of science and technology policymaking) as well as systematic review and PM case studies. The contribution of the study is to provide such a framework for moving toward PM, which has not been addressed from a managerial and policy perspective so far.

## 3 Methods

First, by a systematic review of the literature and the content meta-analysis, the general transition framework to PM was extracted. Then, by case study, the transition framework to PM in Iran was determined according to the different transition phases.

### 3.1 Systematic review

We used PRISMA checklist for systematic review (checklist of preferred reporting items for systematic reviews and meta-analyses) (50), and as much as possible, we followed the standard methods for systematic review to identify relevant literature. We applied a set of inclusion/exclusion criteria: (1) papers were searched based on the keywords “transition” and “personalized medicine,” “personalized medicine” + “transition pathway,” “precision medicine” and “transition pathway,” “transition” and “precision medicine,” or “transition pathway + “precision medicine”; (2) they should be accessible in English; (3) “the outputs should be published in peer-reviewed journals or authentic publications”; (4) the outputs should be relevant to 2010 to 2022^13^; (5) To increase the number of papers, when one article clearly relied on another paper to make a key part of its argument, that paper was also used.

PubMed and ScienceDirect, databases were used for systematic review. The most important reason for using PubMed was that PM is a very specialized subject in health and medicine, and it was easier to find related papers in PubMed. The search strategy was as follows: first, based on the keywords, papers were found, and duplicate articles were removed. In the next step, two of the authors (S.K. and F.S.) independently selected papers according to the titles and topics. Then they excluded some of these studies due to not having access to the full text or being irrelevant according to the abstract. In the subsequent screening, some papers were excluded due to not appropriate methodology or not relevance of papers’ content after an overview of full texts. Then the full texts of the selected papers were read by the authors. Finally, papers were selected based on content analysis, which had the most content related to our research.

**Figure 6:**
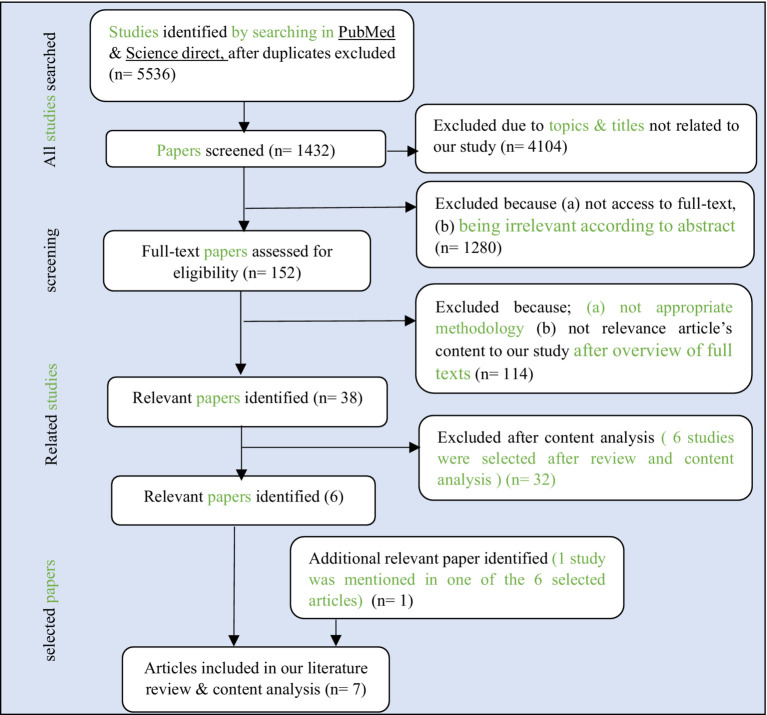
Flow diagram of Systematic review.

Moreover, if there was a disagreement between the two authors, the third person (S, Q.N) was consulted to reach a consensus. Finally, relevant information was extracted from the entered studies. This information included year of publication, names of authors, research area, research method, results and conclusions, and research gap. This information is detailed in Appendix 1. The entered studies were reviewed and analyzed. Figure 6 shows the flow of systematic review.

### 3.2 Case study (qualitative method)

Research scope in Iran’s health sector means the Ministry of Health, international pharmaceutical companies, knowledge-based companies or startups are working about PM, academic research centers, insurance organizations, associations of patients whose PM played a significant role in their treatment, and patients. The statistical population of the research was PM specialists and researchers in Iran and abroad. Moreover, patients whose PM plays a significant role in their diagnosis and treatment and themselves are somewhat familiar with PM and management issues were selected with the previous group.

The sampling method was non-random with a combination of purposive and snowball techniques. It means according to the goal of the researcher, purposive sampling was used, and following it because the specialists familiar with PM and ST could not be easily identified, the snowball technique was used. That is, the authors continued collecting data and interviewing specialists as long as the new data did not lead to more knowledge and formulation of the category. The semi-structured interview was used to collect data (in order to determine the characteristics of TPs and finally the transition framework to PM).

27 samples were selected and interviewed. The interviewees were experts and specialists in the health sector who had knowledge and awareness about PM or have worked in this field for at least 5 years (11 medical geneticists (41%), 6 General and specialized pharmacist (22.2%), 3 general practitioners (11%), 2 nutritionists (7.4%) and other 3) people proteomics specialist, clinical biochemist, 1 biomedicine specialist; 1 Bioinformatics specialist and the expert in healthcare management. The average time of each interview was between 1and 2 h and the average number of interviews was twice for each person (because of the lengthening of some interviews, those people were interviewed 2–3 times to answer all the questions).

The questions were designed based on the characteristics of the ST paths and the initial framework taken from the systematic literature review. Data collection continued until the theoretical saturation of the categories. After the accomplishment of data collection and analysis, two specialists were asked to revise the model or framework resulting from the case study. In order to measure the reliability of the interview tool, the codes extracted from the interviews and the data taken from the content analysis were given to another researcher and the coding was done again. The Kappa index in SPSS software was 0.6975 (above 60%), which is valid. Therefore, the tool used to extract the codes has been reliable. Theme analysis technique and coding was used to analyze the data from the interview. The period of data collection from case study was from September 2022 to March 2023. Some of the codes related to question 1 were:

- Transition from common medicine to stratified medicine and then to PM
- Direct moving from common medicine to PM
- impossibility of moving toward PM in the current situation
- simultaneous start of stratified medicine and PM

The analysis of these codes according to their repetition rate finally led to the identification of the characteristics of the transition paths in the phases of PM development. Because each of the transition paths have different characteristics, which are briefly shown in Table 1. After analyzing the codes, summarizing them, drawing the initial framework, an expert panel was held with presence of two thirds of the interviewees at the end of March 2023.

The researcher, while presenting a report of the analyzed data from the interviews and the initial framework obtained for the transition to PM (this framework was extracted from the systematic review and the results of the interviews), asked the experts to give their opinions and agreement or disagreement regarding the results obtained and the framework for the transition to PM. Finally, after discussing and expressing the positive and negative opinions, the members reached a consensus with the majority voting regarding the framework for the transition to PM.

### 3.3 AHP questionnaire (quantitative method)

In order to clarify the answers to some interviews’ questions and qualitative themes, especially the questions whose answers should be determined according to priority and transition phases, an AHP^14^ questionnaire was designed. It was given to the members of the expert panel to complete based on the researcher’s report and explanation. For example, about this interview’s question, ‘What is the governance style or the space of action in the transition to PM?’, it was presented in the Table 2. The themes of the interview regarding this question were: government and Ministry of Health, civil society/people, and market leadership.

**Table 2:** Importance of supply chain (supply and demand) in transition to PM in early phases.

Scale i	Priorities																	Scale j
Government	9	8	7	6	5	4	3	2	1	2	3	4	5	6	7	8	9	Market
Government	9	8	7	6	5	4	3	2	1	2	3	4	5	6	7	8	9	Civil society/people
Market	9	8	7	6	5	4	3	2	1	2	3	4	5	6	7	8	9	Civil society/people

**Figure 7:**
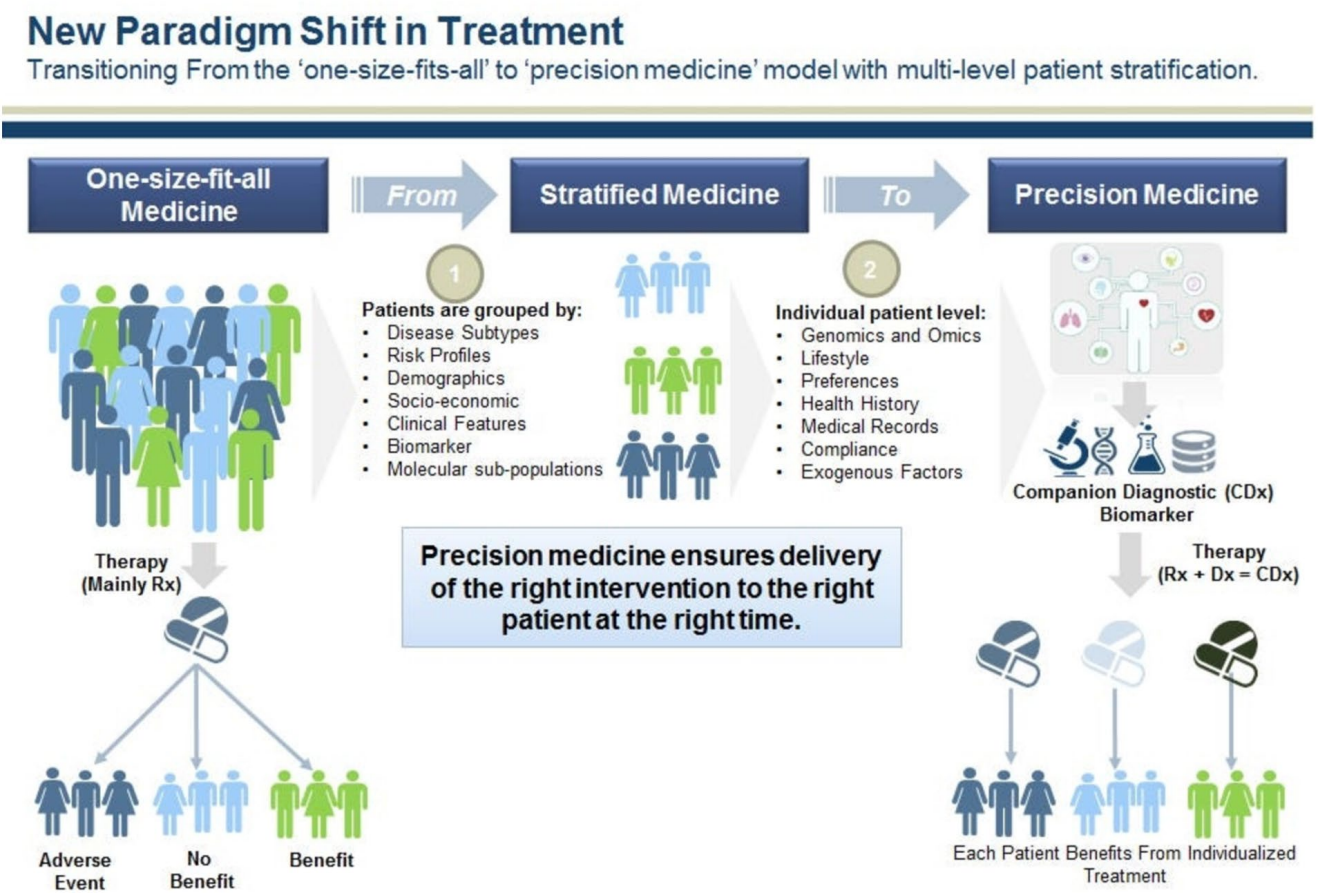
New paradigm shifts in treatment. Source: (43, 44) (Frost & Sullivan Co.).

The data obtained from this questionnaire was entered and analyzed in Expert choice software. Finally, the results obtained from the analysis of AHP questionnaires were added to the results obtained from the interviews and expert panel. These results were in line with and complementary to the results of the interviews and expert panel, and they specified the priority of each of the characteristics related to the TPs in different phases (Figure 7).

## 4 Results

### 4.1 Results from the systematic review

Among the 7 selected articles, the statistical population of 3 articles was related to US, 2 articles related to the Netherlands, 1 article related to the UK, and 1 article related to France. The summary of the results of the systematic review is given in Appendix 1 (Summary table).

**Figure 8:**
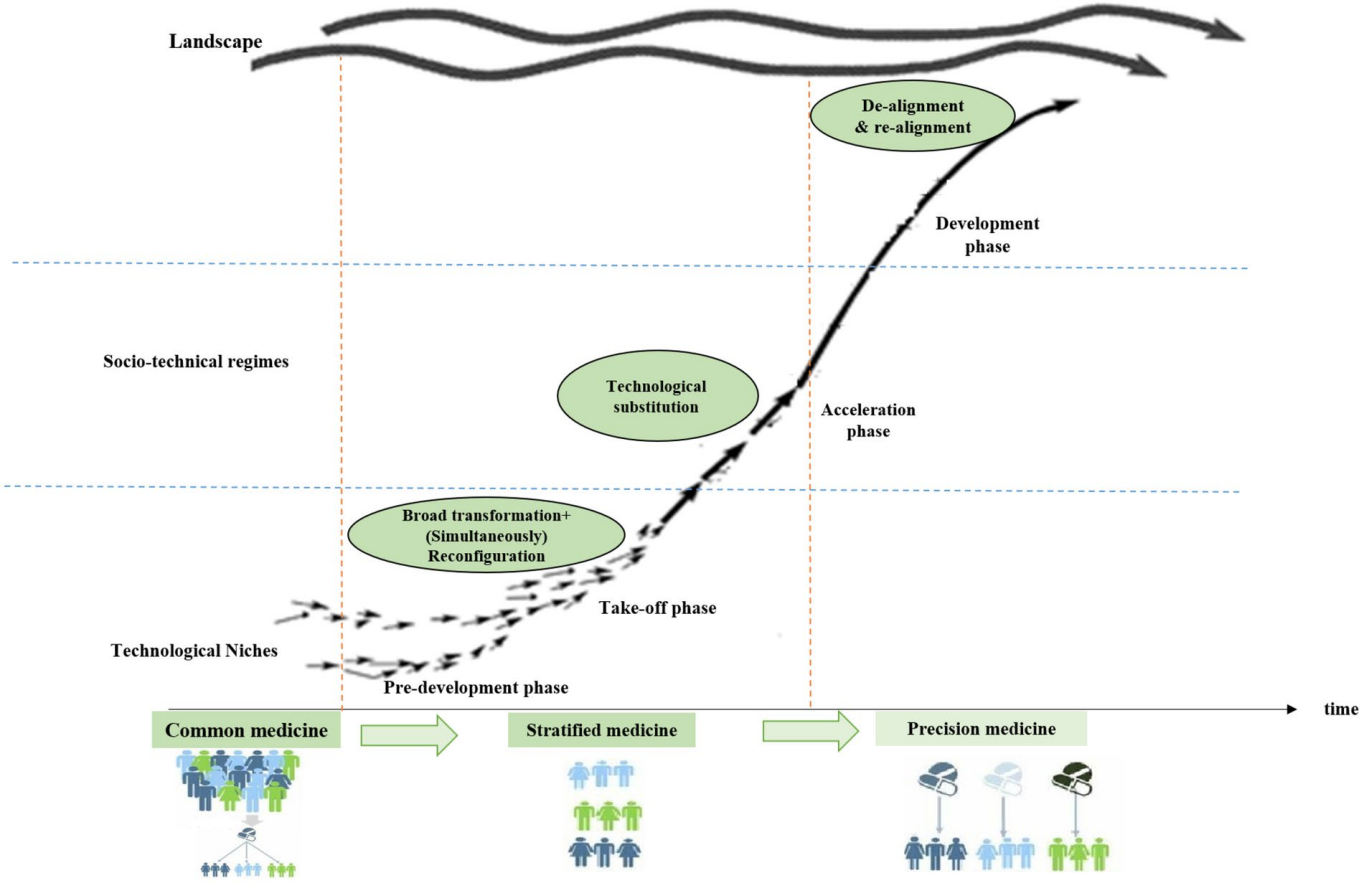
The initial conceptual framework of the transition to PM. Source: the authors.

Finally, among the selected articles, the model presented by Das (43) and Glady (44) was used in this study. The model is shown in Figure 8. In the model, the framework or paths of ST are not mentioned, and from the point of view of transition management or policymaking, has not been paid attention to the subject, but it has mentioned the changes in medicine and health sector in order to achieve PM. Therefore, this model can be used as a general model along with other ST models to design and complete the transition framework to PM.

The possible characteristics of the ST paths to PM in the transition phases are shown in Table 3. These characteristics are based on a systematic review and case study of ST paths and the repetition and similarity of the characteristics in different case studies. In other words, they are based on content analysis of related papers to the transition to PM (authors’ study). According to these characteristics, the authors have defined the following propositions for ST paths to a phenomenon, which is PM here:

- Proposition 1: In the pre-development and take-off phases, the possible socio-technical TP is mainly a combination of broad transformation simultaneously with reconfiguration, which in transition to PM can be considered equivalent to the transition from common medicine to stratified medicine (authors’ study). It is because of the resistance of the health regime in this pathway and the gradual change of the current companies (mainly pharmaceutical and knowledge-based companies) through adopting new practices and models. Also in this pathway, the niche technologies are emerging, landscape pressure is high, the main governor (action space) is the central government, and there is little coordination between actors.
- Proposition 2: In the acceleration and development phases, the possible socio-technical TP are technological substitution and de-alignment and re-alignment, which can be considered equivalent to the transition from stratified medicine to PM (authors’ study). Because of these phases, knowledge-based pharmaceutical companies (niche innovations) have matured and developed to replace common medicine (current regime). The “technology pressure” and actors’ pressure cause the dominant regime to be gradually replaced by emerging PM technologies (new regime) and lead to a radical change in the dominant regime. In this pathway, dominant pharmaceutical companies will either be destroyed by new companies or they will be replaced by innovative companies. Substitution or replacement is often required to change dominant technologies to innovative or radical technologies. In this phase, the main governor is the market, and the supply of PM-based services becomes more important.

**Table 3:** The characteristics of general transition pathways to PM.

The characteristics of transition pathways to PM	1st & 2nd transition phases (pre-development & take-off)	3rd & 4th transition phases (acceleration & stabilization)
Landscape pressure	High	Low
Maturity of niche technology	Low (emergent)	Moderate (mature)
Niche-regime co-existence	Absent	Present
Resilience of regime	High (resilient)	Low (fragile)
Degree of change	Incremental - modular	Radical - architectural
Degree of changes in regulations & institutions	Incremental	High
governance Pattern or action space	Government	Market-led
Importance of value change	Demand is more important	Supply is more important
Incumbent actors’ fate after transition	Majority	Minority
MLP levels involved	Landscape, regime	Landscape, regime & niche
Co- ordination	Low	High
Transition mechanism	Absorbing and personalizing niche technologies	Clustering and empowering of niches & absorption of external innovators
Selected transition pathway to PM (Based on most matching features)	broad transformation+)Simultaneously(reconfiguration	Technological substitution De-alignment and re-alignment

Source: The authors (the results from systematic review).

If the combined TP, i.e., broad transformation simultaneously with reconfiguration (transition in limbo), the appropriate TP from common medicine to stratified medicine, it is expected initially, infrastructures development and regulation instrument, and then stimulating the innovation demand side should be the best technology and innovation policy tools in the early phases of transition because these pathways require government intervention and innovative and knowledge-based companies and accelerators in PM. But in the transition from stratified medicine to PM (technological substitution and de-alignment and re-alignment pathways), stimulating the innovation supply side as a technology and innovation policy tool is more effective.

### 4.2 The general framework for the transition to PM

Since there was no research that explicitly specified the pathways/framework of the transition to PM, the models, or patterns and topics arising from related studies (like pieces of the puzzle) were connected, and the following initial conceptual framework was drawn for the transition to PM. This framework is adapted from the New paradigm shifts in treatment (22, 43, 44), theory of ST pathways by Geels, Scott and Conger (56); (24, 28)] and transition phases model (8, 28) and connecting them together.

### 4.3 Results from the case study and AHP questionnaire

A case study is a suitable research strategy for validating the initial framework of the transition through drawing out the sequencing actions taken by key agents in recognizing and legalizing innovative and entrepreneurial opportunities.

By the case study and results from the interviews, more precisely according to the results of the first question of the interview and AHP questionnaire (Does the transition from common medicine to PM require moving from common medicine to stratified medicine and then PM, or will this transition path be followed directly?), it was determined that the transition path to PM is gradual, and first we should move from common medicine to stratified medicine, and then PM. PM is a step further than stratified medicine; toward personalization is the fifth industrial revolution. The analysis of relevant codes according to their repetition rate finally led to the identification of the transition path. After analyzing the codes obtained from the interviews, summarizing them, drawing the initial framework of transition, and finally implementation expert panel and consensus (based on the lived experience of experts or interviewees) about it, the framework of the transition to PM was drawn as it pass from stratified medicine. So, our study result is in accordance with the initial conceptual framework of the research.

In order to determine the TPs from common medicine to stratified medicine and then to PM, questions were asked based on the characteristics of the TPs (Table 1). Summarizing and integrating the results of the interviews, the expert panel, and AHP questionnaire (the identified characteristics of the TPs to PM in different phases of transition, as well as the selected TPs based on these characteristics) are shown in Table.

**Figure 9:**
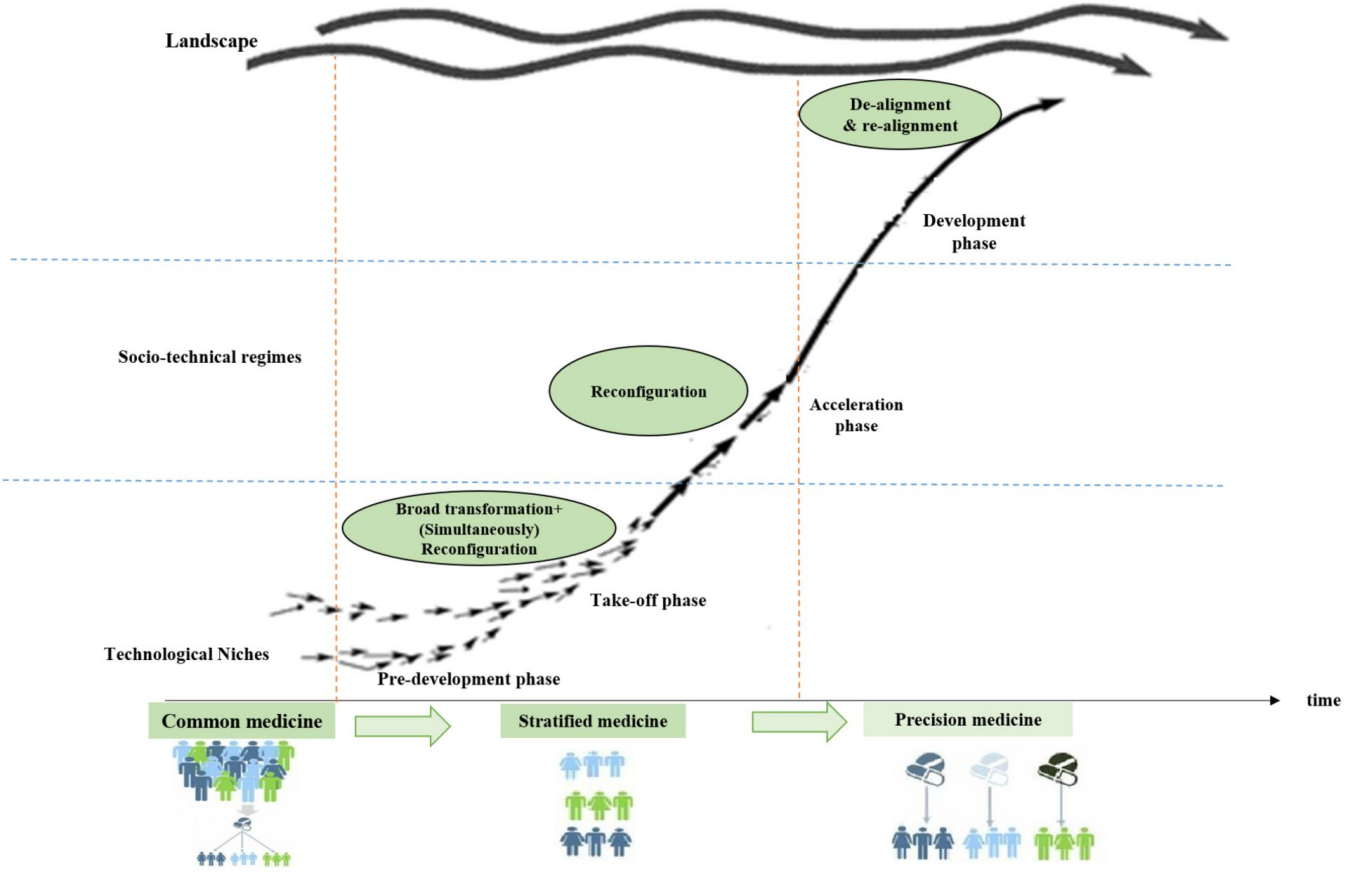
The transition framework to PM in Iran. Source: the authors.

Finally, the transition framework to PM in Iran, which it was the result of a systematic review and content analysis, was drawn. It is shown in Figure 9.

## 5 Discussion

The transition framework to PM in Iran (Figure 9) is largely in accordance with the general framework of PM (Figure 9). In the general framework to PM, in the acceleration phase, the path of technological substitution or reconfiguration is defined, and in Iran, this pathway is reconfiguration. Perhaps the reason for the impossibility of the technological substitution path in the acceleration phase in Iran is due to the high resistance of Iran’s health regime, which itself is caused by the political, economic, and social structure of the country and the governance of the central government, and these are important characteristics of the reconfiguration path. But the other parts of the framework for PM in Iran are in accordance with the general framework for transition to PM (Table 4).

**Table 4:** The characteristics of transition pathways to PM in Iran.

The characteristics of transition pathways to PM	1st & 2nd transition phases (pre-development & take-off)	3rd & 4th transition phases (acceleration & stabilization)
landscape pressure	High	Moderate
Maturity of niche technology	Low (emergent)	Low (emergent)
Niche-regime co-existence	Absent	Absent
Resilience of regime	High (resilient)	High (resilient)
Degree of change	Incremental - modular	Radical - architectural
Degree of changes in regulations & institutions	Incremental	Incremental
governance Pattern or action space	Leadership + Government	Government + Civil Society
Importance of value change	Supply is more important	The same importance of supply & demand
Incumbent actors’ fate after transition	Majority	Majority
MLP levels involved	Landscape, regime & niche	Landscape, regime & niche
Coordination	Low	High
Transition mechanism	Absorbing and personalizing niche technologies	Absorbing and personalizing niche technologies
Selected transition pathway to PM	broad transformation +)simultaneously) weak reconfiguration	Reconfiguration De-alignment and re-alignment

The best TPs to PM in Iran (according to their characteristics) are combined TPs, i.e., broad transformation simultaneously with reconfiguration in the early phases (pre-development and take-off), and reconfiguration and de-alignment and re-alignment in the later phases of transition (acceleration and development).

The results show that PM in Iran is currently in the pre-development phase, and the best proposed transition path to PM in Iran in current conditions is the combined transition path, and at the top of it, the broad transformation path.

Identifying the framework and paths of transition to PM will be a suitable tool for macro policies to develop it in the country. Because by identifying the characteristics of the transition paths toward PM, we can predict the requirements, infrastructures, and challenges of each phase of PM development. For example, is the resistance of the health regime against PM high or low? Is there coexistence between PM start-up companies (niche markets) and the health regime? Are radical and fundamental changes in laws and institutions necessary? The answer to each of these questions leads to the right decision-making by policymakers. It also helps policymakers to know which PM technologies to invest in, what drugs to import or manufacture, and identify and support PM startup companies that are in the R&D phase. The framework identified for the development of PM in Iran can be used in other countries, that their responses to the questions, like in Iran, are also similar to Iran in terms of economic, social, technological, and medical infrastructures, particularly PM technologies and AI. For instance, Turkey, Saudi Arabia, Qatar, the United Arab Emirates, and even India, the latter of which is more advanced than Iran in PM. Because these countries have begun the initial phase of PM development (the pre-development phase of transition growth phases), which is the launch of a genomic data bank of the resident population, or, in other words, Real Data World (RWD).

In addition to these, in the study of Iran, the requirements and challenges of the transition to PM were also identified. The requirements for transition to PM based on importance are respectively: educational, policymaking, technological, infrastructural, scientific-research, legal (regulatory), coordination, executive, actors, demand, insurance, learning, financial and logistic requirements. The challenges of transition to PM are, respectively: policymaking, insurance, patient resistance, financial, infrastructure, actors, coordination, technological, and ethical challenges.

## 6 Conclusion

According to the results of the systematic review as well as the case study, in order to transition from common medicine to PM, we must first experience stratified medicine. In other words, first we should move from the common treatment for the entire population toward the common treatment for a group of patients with the same conditions and then move toward PM. Because the transition to PM requires powerful economic, social, political, institutional, and industrial infrastructures and, most importantly, the acquisition of new PM technologies, the technologies are not quickly achievable even for advanced countries, and it requires steps that can be classified as “stratified medicine.”

According to the general results of the systematic review, the TPs to PM in the order of the transition phases include the combined TP, i.e., broad transformation simultaneously with reconfiguration in the early phases (pre-development and take-off), technological substitution/or reconfiguration (acceleration phase), and de-alignment and re-alignment (development phase).

Most of the research related to PM has dealt with this issue from a specialized, clinical perspective and the PM technologies, which are mainly artificial intelligence and genomics technologies. Moreover, no studies have been seen about PM policymaking and PM management, or particularly the transition to PM, that are comparable to this study. However, some studies can be compared to some extent, such as the following:

The results of our study and the proposed transition framework to PM in the study are consistent with the framework to change the treatment paradigm from common medicine to PM that it has presented by Das (43) and Glady (44).

Whitsel et al. (45) stated that the role of the government should be different in TPs than in PM, and it cannot institutionalize the same policies everywhere. The results of the study are consistent with the results of Whitsel et al. because different policies have been assigned to the different transition paths to PM.

The results show that in order to develop PM, governments should listen to people, technological companies, medicine manufacturers, health service providers, etc., and make policies according to real needs while considering the speed of technological development along with innovation. Policymakers should follow a balance between safety and innovation. These results are consistent with the study of Ghazinoory and Farazkish (46, 51). They should promote the safe use of digital health technologies while establishing regulations for this purpose. They should carefully consider all potential legal implications of using health technologies, including data protection. Moreover, governments should consider educational, policy, technological, infrastructural, scientific-research, legal (regulatory), coordination, executive, actors, demand, insurance, learning, financial, and logistic requirements in the transition to PM and consider solutions for policy challenges, insurance, patient resistance, financial, infrastructural, actors, cooperation, technological, and ethical issues. The studies of Ghazinoory et al. (47) and Ghazinoory and Aghaei (48) have almost confirmed these results.

Since the PM is not only the supply of innovation and new technological services and culture change for using and acceptance of PM and the learning of technologies, and the method of providing services must also take place, new studies should be conducted that also consider these dimensions, and generally, research should be done in the framework of the transformation of a socio-technical system.

## Data Availability

The original contributions presented in the study are included in the article/Supplementary material, further inquiries can be directed to the corresponding author.

## Footnotes

^1^ShareDNA in Seattle (a free smartphone application).

^2^Omics-Based” Sequencing Technologies Across Pathways of Car, Genetics Adviser tool (a digital genetic counselling and education support tool) Liquid biopsy.

^3^myDNA Life (broad pharmacogenomics tests).

^4^PM artificial intelligence platform to identify high-risk genes in covid-19 using biobank data in U.K.

^5^Epigenetic-based technologies and Precision medicine, customized healthcare and Nanobots in EU.

^6^a whole-of-government 10-year initiative aiming to generate precision medicine data of up to one million individuals.

^7^Saudi Genome Program (SGP) has launched in 2018, which plans to build a gene bank particularly for Saudi Arabians.

^8^The Indian Genome Variation database project was started as early as 2003 and reintroduce as DNA technology bill in 2019.

^9^ https://www.researchandmarkets.com/research/n5kqz7/global

^10^ https://www.personalizedmedicinecoalition.org

^11^ https://www.healtheuropa.com

^12^Complex social systems (sub-systems) include transportation, providing energy, housing, and agriculture, healthcare and so on (8, 18, 49).

^13^In order to review the most recent studies and because the history of policymaking in PM in the world is less than 10 years, this period was chosen. It initiated under President Obama’s announcement in 2015 about PM & “all of us” program which emerged from a blue-ribbon working group process involving scientists, patients, physicians, ethicists, and government officials.

^14^ Analytic_hierarchy_process
