# Supplementary material for: A national framework for transition to precision medicine
Source: Front Med (Lausanne). 2025 Jan 27;11:1396496. doi: 10.3389/fmed.2024.1396496 (PMC11841404; doi:10.3389/fmed.2024.1396496)
Supplement: Supplementary file 1 [file Data_Sheet_1.pdf]

Appendix 1 (Summary table): The method and results of selected articles

year	Author	region	Method or approach	Results	Research gap
2010	Loorbach & Rotmans	Netherlands, Belgium	Empirical case studies. Investigating transition management over the past 10 years in 4 different cases: Region (in (Parkstad Limburg), Industry (roof'-transition program), Sector (health-care sector) & international transition (in Belgium in the area of sustainable waste and resource management)	The Transition Program in the Care (2007-2010) was initiated by the Dutch Ministry of Health, Welfare and Sports. The program aims to enable the Dutch care sector to fulfil the need for long-term care. Transition management so far mainly focused on the predevelopment phase of transitions.	Not dealing with the socio-technical transition paths and only using the term transition management in the study
2013	Felix	U.S.A	Literature review by HEOR and International Association of Pharmacoeconomics (ISPOR)	Several examples from the past decade have demonstrated that a variety of PM and diagnostic tests are readily available in clinical interventions, and regulators and payers in the USA and elsewhere have embraced this new paradigm. few HEOR studies exist in the field of PM medicine today.	Considering the current medicine paradigm shift to PM and not dealing with TPs. Using the regulatory framework presented in the research to determine the transition framework to PM in our own research.
2018	Horgan & Lal	Netherlands, Belgium	Division of translational researches which are the basis of PM studies into three time stages1- from laboratory to industrial application, 2- from industrial application to the market, 3- from the market to implementation in the healthcare system. Explaining the stages of technology transfer and medicine production and the relationship between technology transfer and medicine production and finally presenting the flowchart of health technology transfer	In order to expand the use of genome-based technologies in the implementation of PM, public health is responsible for 10 essential tasks in 4 dimensions, which is called the public health genomic wheel. These tasks include: enforce laws, provide care, assure competent workforce, evaluate (assurance dimension), health monitor health, diagnose and investigate (assessment dimension), inform-educate/ empower, mobilize community partnerships, develope Policies (policy development dimension) and management system dimension in the center of the cycle	This study explains the stages of transformation and implementation of PM-based technologies well and is more helpful in PM policymaking.
2019	Whitsel et al	U.S.A	literature review based on health cohort research (All of Us Research Program) (AoURP)	Providing policy levers for the government's significant role in promoting PM and public health	Only, the policy levers presented in the paper will help to select the right policy tool for PM in our study.
2021	Boons et al	U.K	qualitative modelling with system dynamics	Impact of Covid-19 on UK food and mobility provision and understanding the transition pathways as a set of interacting chains of events. The study showed how an important event like Covid-19 leads to multiple effects on the existing transition paths and also the emergence of new paths.	The important event or external shock mentioned in the study is PM in our research, which it will affect the current paths of common medicine and increase the legitimacy of government intervention.<br>But in this study, ST to PM is not discussed.

2017	Das	U.S.A	introducing the framework based on several years of experience and research at Frost & Sullivan Company	Introducing a new framework as “New paradigm shifts in treatment”, in which it refers to the transition from "one size fits all" to PM by stratifying patients on several levels.	The model was considered as the initial framework for transition to PM in our study.<br>But in this study, socio- technical transition to PM is not discussed.
2019	Glady	France	The Bio Immune(G)ene Medicine, so-called BI(G)MED- 3 case reports	Personalized medicine & precision medicine are the same. Therapeutic interest and effectiveness of resorting to the regulating potential of the cell itself, the only one able to rebalance a disturbed mode of functioning, when the needed molecular information is provided to it.	It’s adapted Das’s framework (2017) in this study and clinical case reports only were used PM for 3 diseases. So the study has not considered transition to PM.
